# De Novo Assembly Transcriptome Analysis Reveals the Preliminary Molecular Mechanism of Primordium Formation in *Pleurotus tuoliensis*

**DOI:** 10.3390/genes13101747

**Published:** 2022-09-27

**Authors:** Chunxia Wang, Jinkan Zhou, Zijian Cao, Bao Hu, Jing Wang, Jinying Guo, Suyue Zheng

**Affiliations:** School of Landscape and Ecological Engineering, Hebei University of Engineering, Handan 056038, China

**Keywords:** *Pleurotus tuoliensis*, primordium formation, molecular mechanism, RNA-seq

## Abstract

Primordium formation is extremely important for yield of *Pleurotus tuoliensis*. However, the molecular mechanism underlying primordium formation is largely unknown. This study investigated the transcriptional properties during primordium formation of *P. tuoliensis* by comparing transcriptome. Clean reads were assembled into 57,075 transcripts and 6874 unigenes. A total of 1397 differentially expressed genes were identified (26 DEGs altered in all stages). GO and KEGG enrichment analysis showed that these DEGs were involved in “oxidoreductase activity”, “glycolysis/gluconeogenesis”, “MAPK signaling pathways”, and “ribosomes”. Our results support further understanding of the transcriptional changes and molecular processes underlying primordium formation and differentiation of *P. tuoliensis*.

## 1. Introduction

*P. tuoliensis*, also known as Bailinggu, is a precious, rare, and edible fungus with extremely high nutritive, medicinal, and industrial value [1,2]. It contains multi-vitamins, mineral substances, and 18 kinds of amino acids [3]. Additionally, *P*. *tuoliensis* is rich in fungal polysaccharides, and modern pharmacological studies have shown that the fungal polysaccharides have a variety of biological functions, such as antioxidant activities, antitumor activities, and immune activity [4,5,6]. With its delicious taste, nutrients, and other important uses, *P*. *tuoliensis* is becoming more and more popular in China, Japan, and Korea, so it has become widely commercially cultivated [7]. Wild *P*. *tuoliensis* is usually distributed in Xinjiang province [8]. Many agricultural wastes can be used as substrates for *P*. *tuoliensis*, such as cottonseed hulls, sawdust, and corn stalks [9,10].

*P*. *tuoliensis* has been commercially cultivated on a large scale since 1997. By 2012, the output of *P*. *tuoliensis* had reached 300,000 tons. However, the yield is unstable and has gradually decreased in recent years [11]. The development of the *P*. *tuoliensis* industry has been seriously restricted by the small number of cultivated varieties, single main cultivated variety, long cultivation period, and strain degradation [12,13]. The formation of the primordium is vitally important for the yield. The cultivation cycle of *P*. *tuoliensis* requires multiple environmental factors to initiate the primordium, such as humidity, special temperature and light conditions, and so on [14,15]. Temperature can affect the growth of edible mushrooms [16]. Cold stimulation is the main trigger for primordium initiation. Cold stimulation following physiological after-ripening of mycelia for 9–10 d could improve the consistency of budding of the primordium, ensuring a high degree of fruiting body uniformity [17]. Light is necessary for primordium initiation [18,19,20,21]. In the commercial cultivation of *P*. *tuoliensis*, the formation of the primordium is generally induced by alternating day and night light [17]. Many studies on the mechanism of fungi primordia formation have been reported. *FBH1* positively regulated the primordium formation of *P. ostreatus*. The downregulated expression of *FBH1* resulted in fewer primordia [22]. *Pofst3* played a negative role in the primordium formation. The primordium formation of *Pofst3*-overexpressing strains was inhibited [23]. CaCl_2_ could inhibit tripe development and thus affected the primordium formation [24]. However, the molecular mechanisms underlying the formation of the primordium remain unclear.

In recent years, high-throughput sequencing technologies have developed rapidly, and transcriptomics, metabolomics, and other omics have been widely applied to explore the growth and development of edible mushrooms [11,25,26,27]. Studies on *Ganoderma lucidum* [28], *Cordyceps militaris* [29], and *P. tuoliensis* [30] have explored the candidate genes related to fruiting body development. In addition, transcriptomics technology has been used to demonstrate the molecular mechanisms in response to various stresses. Transcriptome data highlighted the *hsp70*, *hsp90,* and *fes1* genes, which were involved in the early stages of fruiting body development in *Flammulina filiformis* [31]. GC-MS and LC-MS in combination were used to study extracellular metabolites of the mycelium, which were subjected to high temperature in *P. ostreatus* [32]. These studies were conducive to studying the growth of mushroom.

Many studies of *P*. *tuoliensis* have been carried out to investigate mycelial maturity [30], the response to cold stimulation in mycelium [17], and the novel genes related to mushroom formation, etc. [33]. However, the research on the formation of the primordium is very limited. A comprehensive understanding of primordium formation is not only conducive to production, but also helpful to reveal mechanisms of development in *P*. *tuoliensis*. In this study, Illumina sequencing technology was used to perform a transcriptome analysis during four primordium formation stages. Twelve cDNA libraries were constructed and differentially expressed genes and pathways were identified. Our results provide new insights into the mechanism of primordium formation. Moreover, the sequences identified here can be used in future marker-assisted breeding programs to speed up the breeding progress of *P*. *tuoliensis*.

## 2. Materials and Methods

### 2.1. P. tuoliensis Cultivation

The *P. tuoliensis* (CCMSSC00489) was obtained from the Agricultural Culture Collection of China. The strain was activated on potato dextrose agar (PDA) medium at 25 °C for 7 days. We selected four different stage samples during the process of primordium formation. Pre-cultured mycelia were inoculated onto the substrates in the culture dishes. These culture dishes were then incubated at 25 °C for 7 days without light (Stage I). We randomly selected fifteen culture dishes of Stage I and placed the mycelia into three tubes on average and froze them in liquid nitrogen for RNA extraction. The rest of the culture dishes were cultivated at 4 °C for 3 days in the dark (Stage II). The mycelia of Stage II were also collected using the same method. In Stage III, the rest of the culture dishes were treated with alternate 12 h light and 12 h dark per day at 16 °C for 7 days to induce primordium formation. The method of collecting mycelia remained unchanged. The rest of the culture dishes were also cultivated under 12 h light and 12 h dark up to primordium formation (Stage IV). The primordia were taken into three corresponding tubes on average and frozen in liquid nitrogen. All samples were frozen at –80 °C.

### 2.2. RNA Extraction

The RNA was isolated separately from each sample using a plant genomic RNA kit (TIANGEN, Beijing, China). The quality of RNA was measured on agarose gels. RNA was checked for purity, concentration, and integrity using the NanoPhotometer^®^ spectrophotometer (IMPLEN, Los Angeles, CA, USA). RNA with a 260/280 nm ratio of 1.8–2.0 was chosen for analysis.

### 2.3. Library Preparation and RNA-seq

A total amount of 1.5 µg RNA per sample saved in each tube was used as the input material for the RNA sample preparations. Three cDNA libraries used as three biological replications were constructed for each stage and twelve sequence libraries were generated using NEBNext^®^ Ultra™ RNA Library Prep Kit for Illumina^®^ (NEB, San Diego, CA, USA). After that, paired-end sequences of the twelve libraries were performed on an Illumina HiSeq 2500 platform by Novogene Co., LT. (Tianjin, China).

### 2.4. Transcriptome Assembly and Gene Function Annotation

Raw data in FASTQ format were firstly processed through in-house perl scripts. Adapter sequences, sequences containing ploy-N, low-quality reads (<Q20), and sequences with lengths less than 50 bp were removed to obtain high-quality clean data by Cutadapt (version 1.15). In addition, the Q20, Q30, GC-content, and sequence duplication level of the clean data were calculated. Clean data were used for transcript and unigenes by Trinity software [34]. The unigenes with length less than 300 bp were removed. All unigenes were pooled to yield the final unigene library. Based on sequence homology, all assembled unigenes were annotated for function based on eight public databases, including Clusters of Orthologous Groups of proteins (KOG/COG), KEGG Ortholog database (KO), Gene Ontology (GO), Protein family (Pfam), Swiss-Prot, NCBI non-redundant protein sequences (Nr), and NCBI non-redundant nucleotide sequences (Nt).

### 2.5. Analysis of Differentially Expressed Genes (DEGs)

Differential expression analysis was performed on each adjacent developmental stage of primordium formation. Analyses were based on three biological replicates per stage. RSEM was used to calculate transcript abundance by mapping clean reads to the unigene database [35]. The DESeq package was used to identify DEGs (*p*-value < 0.05, |log_2_ Fold Change ≥ |1) [36].

### 2.6. Functional Analysis of DEGs

GOseq and KOBAS software [37] were used to analyze the statistical enrichment of DEGs in GO terms and KEGG pathways.

### 2.7. Validation of Transcriptomics Data

qRT-PCR was used to validate the results and 10 DEGs were chosen randomly. The RNA was the same as that used for RNA-seq analysis. Primers were designed by Primer 5.0 software and are shown in Table S1. Glyceraldehyde-phosphate dehydrogenase gene (*GAPDH*) was used to as an internal reference gene [30]. The qRT-PCR analysis was carried out on the ABI 7500 Real-Time PCR System (Applied Biosystems, Los Angeles, CA, USA) with 2×RealStar Green Power Mixture with ROX II (GenStar, Beijing, China). Each reaction contained 50 μL of reaction mixtures (1 μL of cDNA template, 1 μL of Forward Primer, 1 μL of Reverse Primer, 25 μL of 2×RealStar Green Power Mixture, 1 μL of ROX Reference Dye, 21 μL of RNase-Free H_2_O). Amplification conditions were 95 °C for 5 min, 95 °C for 15 s, 60 °C for 1 min, 72 °C for 30 s, three cycles. The relative gene expression levels were calculated using the 2^−∆∆Ct^ method [38].

## 3. Results

### 3.1. The Four Developmental Stages of Primordium Formation of P. tuoliensis

**Figure 1:**
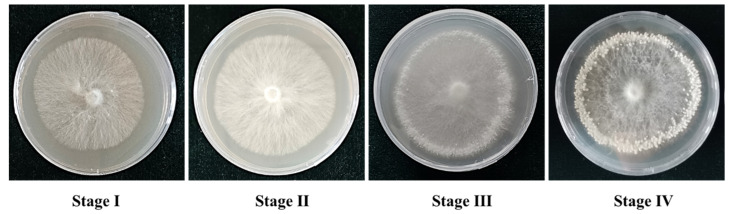
Four stages of primordium formation of *P. tuoliensis*.

The developmental process of primordium formation of *P. tuoliensis* includes four major stages (Figure 1): mycelia growth (Stage I), cold stimulation of Stage I mycelia (Stage II), induction of primordium formation (Stage III), and primordium (Stage IV). The vegetative growth development occurs in Stage I and Stage II, the transition of vegetative to reproductive growth occurs in Stage III, and the reproductive growth development occurs in Stage IV. In the absence of cold stimulation and light, the transition from vegetative growth to reproductive growth rarely occurs. The culture conditions of *P. tuoliensis* were as follows: mycelia were first grown at 25 °C in cultivation dishes for up to 7 days in the dark (Stage I), then cold stimulation of mycelia at 4 °C for 3 days without light was carried out (Stage II). Then, the mycelia were grown at 16 °C in light 12 h/day for 7 days (Stage III). The mycelia were then incubated at 16 °C in light 12 h/day for another 7 days (Stage IV). The mycelia turned into primordia in Stage IV. The environmental conditions required for the formation of the primordium had previously been studied, but the molecular mechanism remained unknown. To investigate the molecular basis of primordium formation, mycelia and primordia of four stages were collected for further analysis.

### 3.2. RNA Sequencing

Twelve cDNA libraries were constructed from the four stages, with three replicates per stage. A total of between 21,091,886 and 23,211,965 raw reads were obtained after Illumina sequencing. After quality control, 76.31 Gb of clean reads was used for further study (Table S2). The Q20, Q30, percentage, and GC percentage are shown in Table S2, and indicated reliabilities of these libraries. A total of 57,075 transcripts and 6874 unigenes were assembled. The N50 length of transcripts and unigenes was 5492 bp and 5535 bp, respectively. The N90 length of transcripts and unigenes was 2128 bp and 1886 bp, respectively (Table S3). The assembly results were validated by busco (Table S4) and the read remapping rate (Table S5). In addition, the PCA analysis showed that biological replicates from the same stage could be clustered together (Figure S1). These results indicated that the data were reliable and reproducible. The raw data were deposited at NCBI SRA under the accession number: PRJNA810571.

### 3.3. Gene Functional Annotation

**Figure 2:**
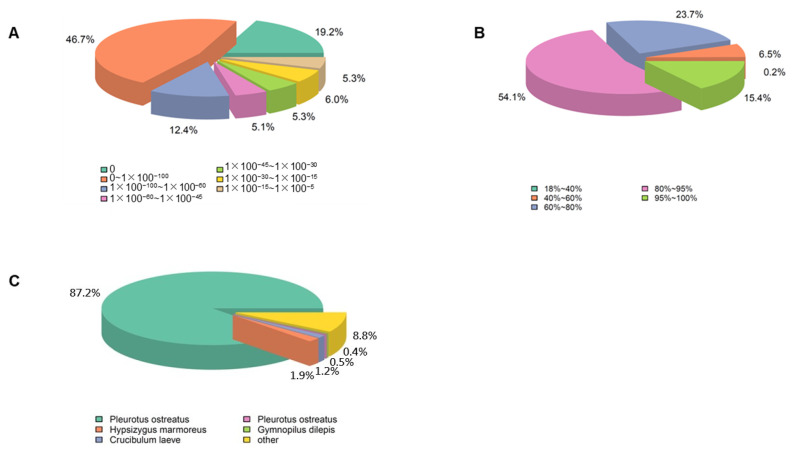
Alignment statistics. (**A**) E-value distribution; (**B**) similarity distribution; (**C**) species distribution.

Twelve cDNA libraries of unigenes were annotated with major databases, including NR, NT, KO, Swiss-Prot, Pfam, GO, and KOG. Proportions of 81.65%, 23.31%, 30.57%, 52.7%, 65.98%, 65.97%, and 35.52% of the unigenes were successfully annotated. In addition, a total of 12.13% of unigenes were annotated in all databases (Table S6). The E-value distribution showed that matches with an E-value of 0 to 1 × 10^−100^ had the largest ratio, which was 46.7% (Figure 2A). As shown in Figure 2B, about 69.5% of unigenes had a strong similarity greater than 80%, whereas 30.2% of unigenes had a similarity value between 40% and 80%. The species distribution analysis showed that 87.2% of unigenes was similar to that of *P. ostreatus* (Figure 2C).

**Figure 3:**
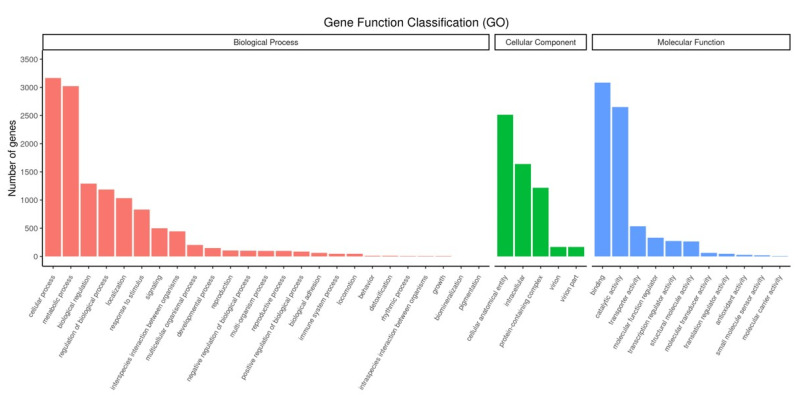
GO terms distribution of annotated unigenes in *P*. *tuoliensis*.

In GO analysis, 4535 unigenes were annotated to three main GO categories. Within the cellular component (CC) category, 55.41% of unigenes were related to cellular anatomical entity, 36.19% to intracellular, and 26.84% to protein-containing complexes. Within the biological process (BP) category, the majority of unigenes were assigned to cellular process (69.81%), metabolic process (66.62%), biological regulation (28.49%), and regulation of biological process terms (26.20%). Moreover, unigenes related to binding (68.00%) and catalytic activity (58.43%) were enriched in the molecular function category (Figure 3).

**Figure 4:**
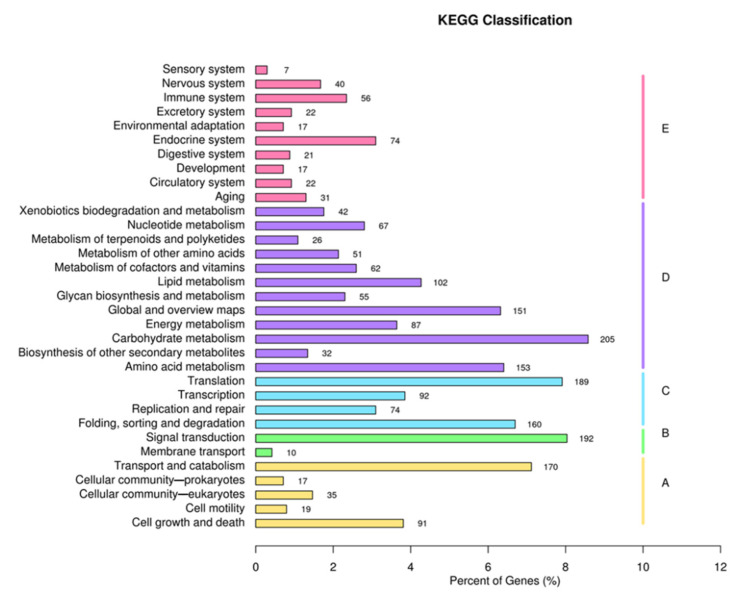
KEGG classification of *P. tuoliensis* transcriptome into functional groups.

KEGG analysis showed that unigenes were represented in five pathways: “Cellular Processes” (332 unigenes), “Environmental Information Processing” (202 unigenes), “Genetic Information Processing” (515 unigenes), “Metabolism” (1033 unigenes), and “Organismal Systems” (307 unigenes). The number of unigenes related to carbohydrate metabolism was the highest (Figure 4).

**Figure 5:**
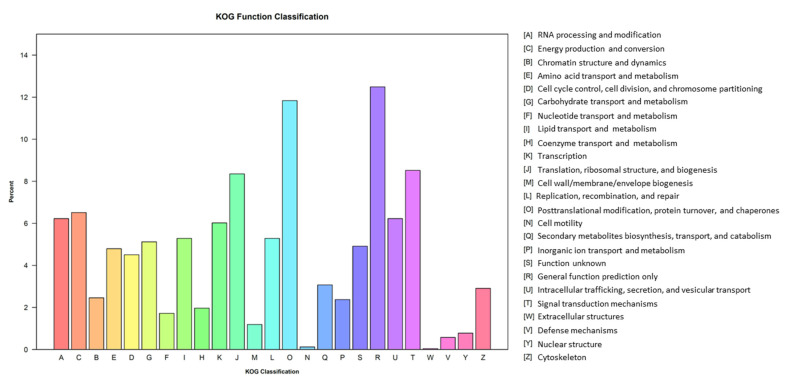
KOG classification of the *P*. *tuoliensis* transcriptome into functional groups.

For KOG annotation, 2442 unigenes were annotated for the *P*. *tuoliensis* transcriptome. These unigenes were assigned to 25 categories. Among them, “General function prediction only”, “Posttranslational modification, protein turnover, chaperones”, “Signal transduction mechanisms”, and “Translation” were the most represented KOG pathways (Figure 5).

### 3.4. Identification of DEGs in Four Stages

**Figure 6:**
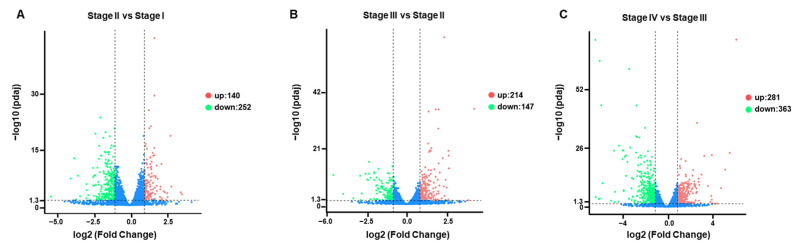
Volcano plots showing the expression level of each unigene. Limits defined by *p*-value ≤ 0.05 and |log_2_ ratio| ≥ 1. Red points represent upregulated genes; blue points represent downregulated genes. (**A**) Volcano plots showing the expression level of each unigene of Stage II compared to Stage I. (**B**) Volcano plots showing the expression level of each unigene of Stage III compared to Stage II. (**C**) Volcano plots showing the expression level of each unigene of Stage IV compared to Stage III.

DEGs were identified to study transcript differences in primordium formation. Overall, there were 140 upregulated DEGs and 252 downregulated DEGs between Stage II and Stage I (Figure 6A), 214 upregulated DEGs and 147 downregulated DEGs between Stage III and Stage II (Figure 6B), and 281 upregulated DEGs and 363 downregulated DEGs between Stage IV and Stage III (Figure 6C). These results indicated the highest number of DEGs in the transition from vegetative mycelium to primordium, suggesting that this period was the most active. Among these DEGs, only 26 unigenes were communally altered in all stages (Table S7).

### 3.5. Functional Enrichment of DEGs

To investigate the biological functions of DEGs involved in primordium formation, GO analysis was performed. Compared to Stage I, upregulated DEGs were enriched in 92 terms (Table S8) and downregulated DEGs were enriched in 80 terms in Stage II (Table S9). In Stage III compared to Stage II, 41 functional terms were upregulated, among which, 5, 19, and 17 of the terms were classified as cellular components, biological processes, and molecular functions, respectively (Table S10). Significantly downregulated terms included 11 cellular component terms, 69 biological process terms, and 31 molecular function terms (Table S11). In the Stage IV:Stage III comparison, DEGs were enriched in 1882 terms, including 62 terms (Table S12) that showed significant enrichment (*p*-value < 0.05). Oxidoreductase activity (GO: 0016705) was enriched in most DEGs, indicating that oxidation–reduction processes may be highly active in primordium formation.

**Figure 7:**
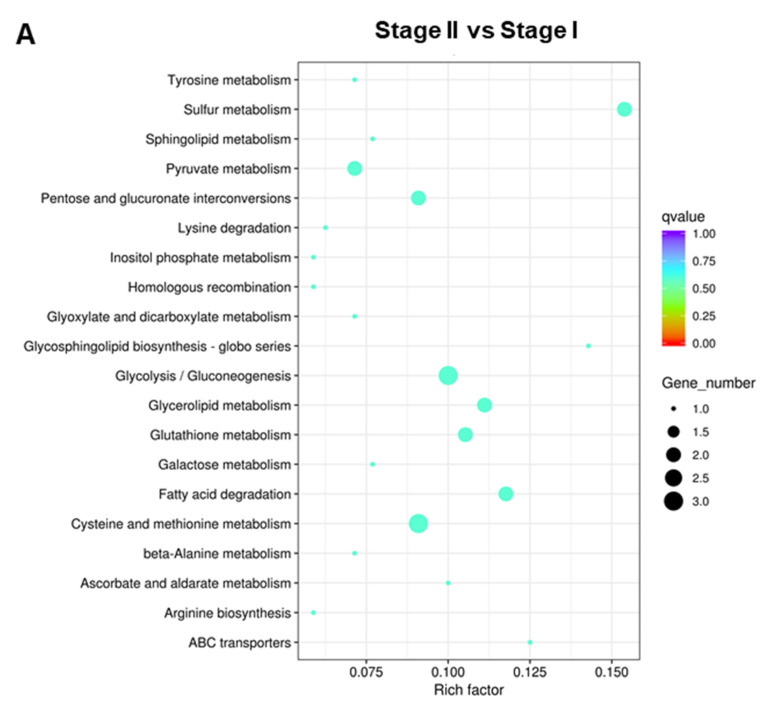

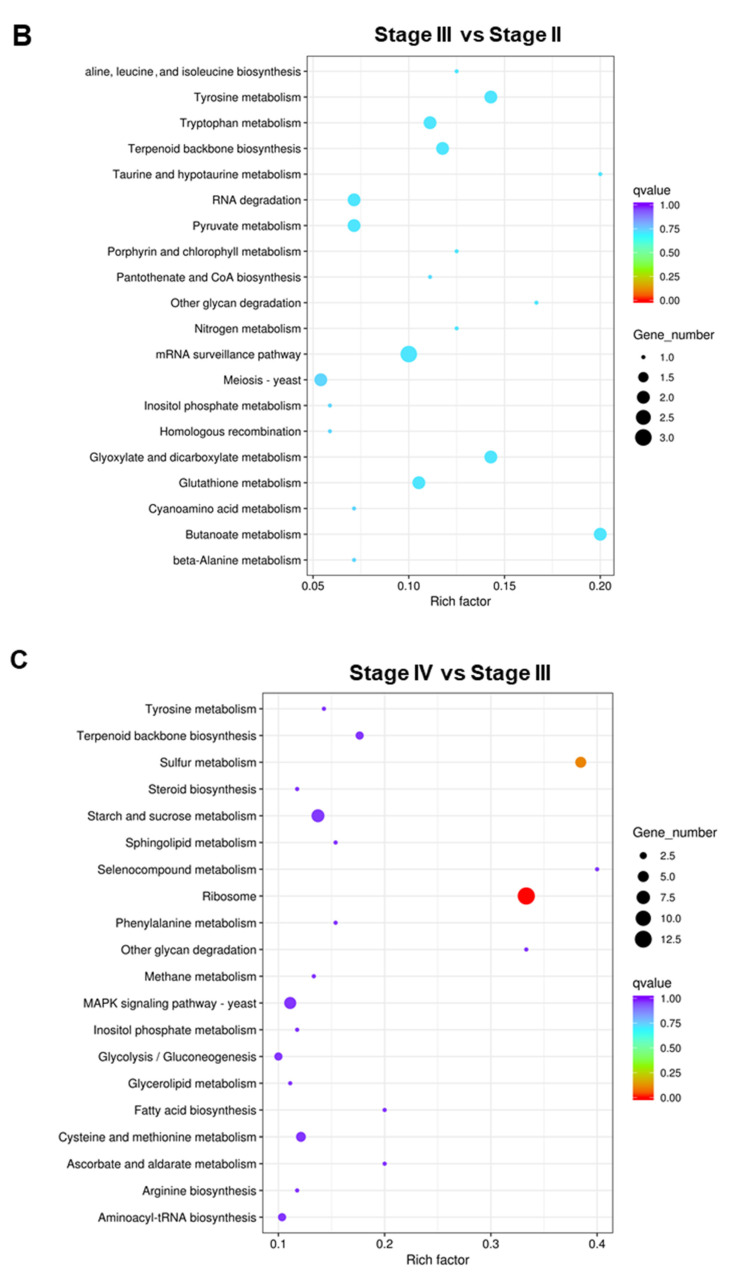
The top 20 enriched KEGG pathways of DEGs. (**A**) Stage II vs. Stage I; (**B**) Stage III vs. Stage II; (**C**) Stage IV vs. Stage III.

To better understand the functions and interactions of these DEGs, KEGG pathway enrichment was also conducted. In the Stage II:Stage I comparison, DEGs were enriched in 39 pathways (Table S13). The top 20 KEGG pathways are shown in Figure 7A. In the Stage III:Stage II comparison, DEGs were enriched in 43 pathways (Table S14). The top 20 KEGG pathways are shown in Figure 7B. In the Stage IV:Stage III comparison, DEGs were enriched in 61 pathways (Table S15). The top 20 KEGG pathways are shown in Figure 7C. DEGs were enriched in “Glycolysis/Gluconeogenesis”, “Cysteine and methionine metabolism”, “mRNA surveillance pathway”, and “Ribosomes”.

### 3.6. Identification of Candidate Genes Involved in Primordium Formation

**Figure 8:**
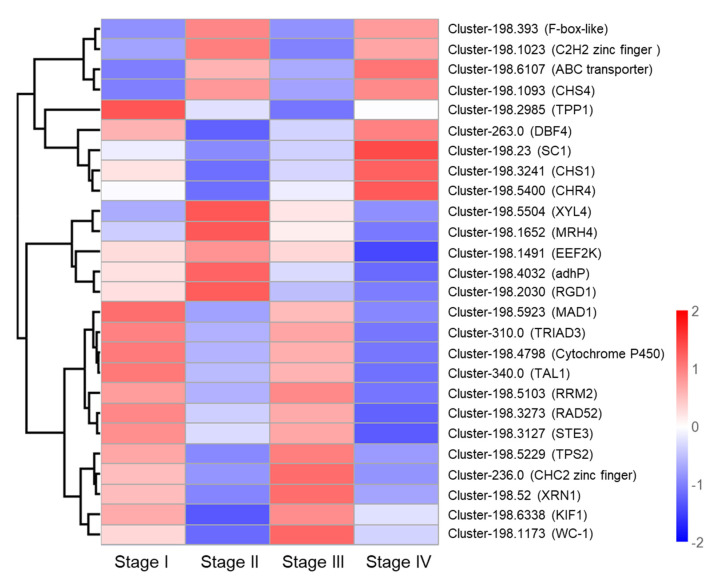
Heat map analysis of 26 DEGs associated with primordium formation.

To identify genes involved in primordium formation, we analyzed 26 DEGs, which were communally altered in all stages. The 26 DEGs were selected for heat map analysis (Figure 8). No gene was continuously upregulated in these four stages. Overall, these DEGs were mainly involved in energy metabolism, protein synthesis, hormone synthesis, and lipid metabolism, demonstrating that primordium formation is a complex process which requires the participation of a variety of regulatory mechanisms. In addition, primordium formation may need ABC transporters. ABC transporters, as one of the largest transporters, can carry out ATP-dependent efflux of substrates [39]. The ABC transport gene (Cluster-198.6107) was significantly upregulated in Stage IV. Post-modification enzymes may play an important role in light response. The expression of Cluster-198.4798 encoding Cytochrome P450 monooxygenase was found to be significantly upregulated in Stage III. CHC2 zinc finger TF (Cluster-236.0) may negatively regulate the response to cold stress, and was found to be significantly downregulated in Stage II.

### 3.7. Validation of DEGs by qRT-PCR

**Figure 9:**
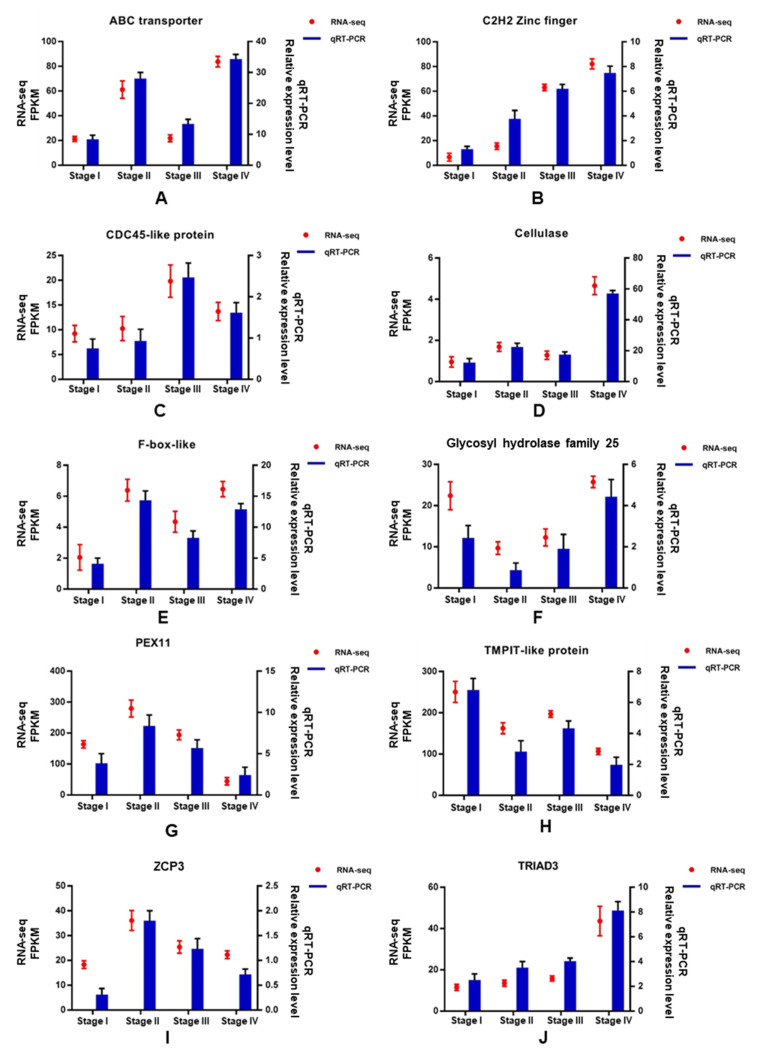
qRT-PCR results of 10 DEGs.

To verify the accuracy of transcriptome sequence data, 10 unigenes were randomly selected for quantitative RT-PCR (qRT-PCR), including ABC transporter (Cluster-198.6107), C2H2 Zinc finger (Cluster-198.616), CDC45-like protein (Cluster-198.5286), cellulase (Cluster-198.720), F-box-like (Cluster-198.215), Glycosyl hydrolase family 25 (Cluster-198.6122), PEX11 (Cluster-198.3467), TMPIT-like protein (Cluster-198.3145), transcription factor ZPC3 (Cluster-198.6036), and TRIAD3 (Cluster-198.1396). As shown in Figure 9, the trend of expression changes of these selected genes based on qRT-PCR was similar to those detected by the RNA-seq method, suggesting that the transcriptome data were reliable.

## 4. Discussion

The formation of the primordium is vitally important for the development of the fruiting body and the yield of edible fungi. The production cycle of *P*. *tuoliensis* is longer than other *Pleurotus* species, especially the formation of the primordium, which lasts approximately 70–100 days [40]. The long cultivation period has restricted industrial development of *P*. *tuoliensis*. A previous study was carried out to identify genes associated with the fruiting body. Fu et al. found that DEGs involved in morphogenesis, primary carbohydrate metabolism, cold stimulation, and blue-light response were related to Bailinggu mushroom formation [33]. A comparative transcriptome analysis of immature and mature mycelia to investigate physiological maturation properties of *P*. *tuoliensis* was performed by Du et al. Transcriptomic analysis revealed that nucleotide synthesis and energy metabolism were highly active during the physiological maturation of mycelia. NDPK was predicted to be essential for mycelia maturation [30]. GC-MS-based metabolomics was performed to study metabolic changes during mycelium physiological maturation of *P*. *tuoliensis*. The content of CA-asp may be an indicator for mycelial maturation [11]. However, no transcriptome analyses have been performed to research the molecular mechanisms underlying the formation of the primordium. In this study, comparative transcriptome analysis was performed to identify genes involved in primordium formation, which comprised the important life stages of *P*. *tuoliensis,* including the mycelia, vegetative mycelia, one hyphal knot, and one primordium stages.

Primordium formation is a complex physiological process. The vegetative growth development occurred in Stage I and the rapid growth of mycelium required more nutrients and energy. Cold stimulation of mycelia occurred in Stage II. GO and KEGG pathway analysis found that these DEGs of Stage II were associated with gluconeogenesis and protein metabolism pathways. Light stimulation is necessary for primordium formation. *White collar 1* (*WC-1)* was upregulated in Stage III. Genes related to cell wall function were significantly upregulated in Stage IV, including *CHS1* and *CHS4*. In Stage IV, we also found oxidoreductase activity enriched with most DEGs. We speculated that genes related to oxidation–reduction activities may also play a vital role in primordium formation.

Transcription factors that are essential for the development of fruiting bodies in fungi have been identified. In *Schizophyllum commune*, the deletion of the transcription factors *HOM1* and *GAT1* resulted in the formation of more but smaller fruiting bodies with an unusual morphology compared to the wild type [41,42]. The transcription factor C2H2 played an important role in fruiting body formation in *Agaricus bisporus* [43]. Liu et al. found that three TFs (Zinc finger, C2H2 type, Zinc finger, Ring type, and Zn (2)-C6 fungal type) were upregulated genes, especially in primordium development, which reinforced the role of these TFs in complex multicellularity in *Flammulina filiformis* [31]. In this study, we found only one transcription factor (Zinc finger, CHC2 type) in 26 DEGs, which were differentially expressed during all four stages. The expression of this transcription factor was highest in Stage III, indicating that it may be essential for the blue-light response rather than the cold stress response. Future efforts will focus on the function of the transcription factor CHC2 and the mechanism by which it regulates primordium development. In addition, we also found other transcription factors, such as C2H2, bHLH, and TFB2, among all of the DEGs. This indicates that the formation of the primordium is a process that requires the participation of a variety of regulatory mechanisms, and transcription factors may play a vital role.

Many KEGG pathways are associated with the development of fungi, such as carbon metabolism [44], oxidative phosphorylation [20], the cell cycle [45], RNA transport [46], and MAPK signaling pathways [47,48]. Functional enrichment analysis of our DEGs found that a large number (13) of ribosomal protein encoding genes were involved in the reproductive growth stage compared to the vegetative growth stage (Stage IV to Stage III). In *Flammulina filiformis*, ribosomal protein encoding genes were hub genes in the young fruiting body. The results indicated that more protein is needed during reproductive growth [31]. The TCA cycle pathway might play a crucial role in providing energy during fruiting body development [33]. Similarly, more energy was required for the formation of the primordium, and the cells employ starch and sucrose metabolism to a greater extent in Stage IV. Unigenes in MAPK signaling pathways are involved in cell growth and differentiation in *Coprinopsis cinerea* and other fungi [46,49]. However, we observed that unigenes related to MAPK signaling pathways were downregulated in the formation of the primordium. This might indicate that the mechanism of primordium formation is different from that of the fruiting body.

Light is the key signaling element for every living cell [50]. In fungi, blue light is a main environmental signal. Blue light can regulate stipe elongation, cap formation, mycelium browning, and so on [51,52,53,54,55,56,57]. A comparative transcriptome analysis that identified candidate genes involved in mycelium browning in *Lentinula edodes* was performed by Yoo et al. This study systematically researched the expression patterns of light-induced browning-related genes, which were significantly associated with light sensing via photoreceptors such as FMN- and FAD-bindings [58]. A comparative transcriptomic analysis of pilei grown under blue- and red-light irradiation in *Pleurotus eryngii* revealed DEGs were significantly associated with light sensing, signal transduction, cell wall degradation, and melanogenesis [59]. In this study, we also found that blue light was essential for the formation of the primordium. We identified genes involved in blue-light response, such as genes encoding the cytochrome, photoreceptor, SC3 hydrophobin, and white-collar 2, which have been reported to be associated with the growth and development of edible fungi.

## 5. Conclusions

This study broadened our knowledge of the molecular mechanism of primordium formation by comparative transcriptome analysis of four stages of growth in *P*. *tuoliensis*. The formation of the primordium is a very complex process, which involves functions related to, e.g., the cell wall, ribosomal proteins, light sensing, cold stimulation, oxidoreduction, and carbohydrate metabolism. In this work, 1397 DEGs were identified, including ribosomal protein encoding genes, white-collar 2 type of transcription factor genes, cytochrome encoding genes, MAPK signaling pathway genes, and other metabolism related genes. Among these DEGs, 26 DEGs were communally altered in all stages, which played an important role in many pathways, such as protein phosphorylation, transcription, translation, the oxidation–reduction process, and so on. These data provided a valuable resource for further studies on *P*. *tuoliensis*. Further studies should focus on the functional features of the 26 DEGs to enable better understanding of primordium formation and provide a foundation for future breeding.

## Data Availability

Not applicable.

## Supplementary Materials

The following supporting information can be downloaded at: https://www.mdpi.com/article/10.3390/genes13101747/s1, Figure S1: PCA reduction in gene expression profiles in three biological replicates from Stage I (yellow), Stage II (red), Stage III (green), and Stage IV (blue); Table S1: Primers used for qRT-PCR; Table S2: Information about sequencing of *P. tuoliensis*; Table S3: Information about transcriptome assembly; Table S4: The statistical results of BUSCOs; Table S5: The statistical results of read remapping rate; Table S6: Information about gene functional annotation; Table S7: List of 26 DEG expression profiles in different stages; Table S8: The enriched GO terms of upregulated DEGs of Stage II compared to Stage I; Table S9: The enriched GO terms of downregulated DEGs of Stage II compared to Stage I; Table S10: The enriched GO terms of upregulated DEGs of Stage III compared to Stage II; Table S11: The enriched GO terms of downregulated DEGs of Stage III compared to Stage II; Table S12: The enriched GO terms of DEGs of Stage IV compared to Stage III; Table S13: The enriched pathway terms of Stage II compared to Stage I; Table S14: The enriched pathway terms of Stage III compared to Stage II; Table S15: The enriched pathway terms of Stage IV compared to Stage III.
