# Supplementary material for: De Novo Assembly Transcriptome Analysis Reveals the Preliminary Molecular Mechanism of Primordium Formation in Pleurotus tuoliensis
Source: Genes (Basel). 2022 Sep 27;13(10):1747. doi: 10.3390/genes13101747 (PMC9601356; doi:10.3390/genes13101747)
Supplement: Supplementary file 1 [file genes-13-01747-s001.zip › Supplementary materials/Figure S1.pptx]

## Slide 1
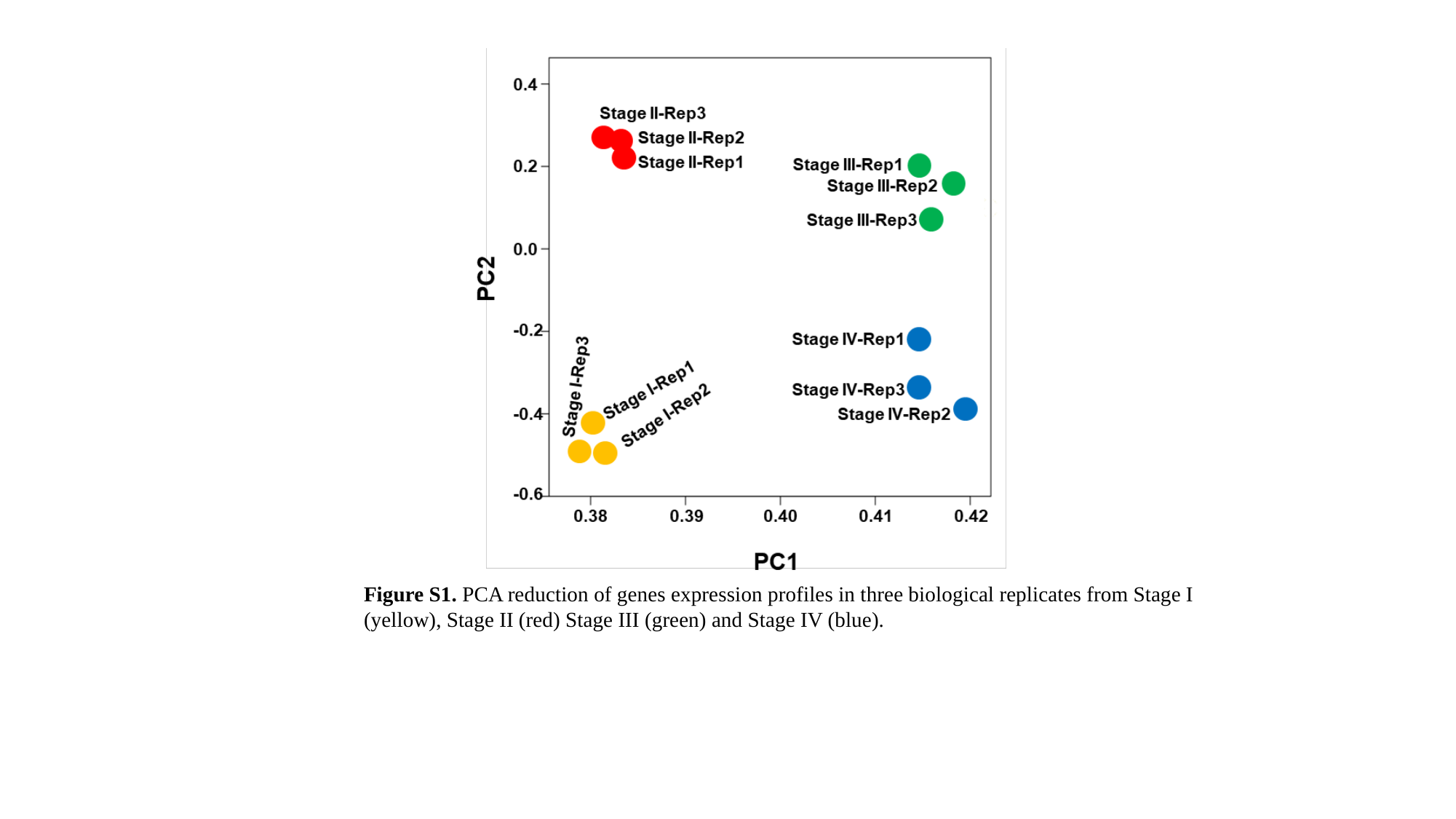

Figure S1. PCA reduction of genes expression profiles in three biological replicates from Stage I (yellow), Stage II (red) Stage III (green) and Stage IV (blue).
